# Economic Linear Parameter Varying Model Predictive Control of the Aeration System of a Wastewater Treatment Plant †

**DOI:** 10.3390/s22166008

**Published:** 2022-08-11

**Authors:** Fatiha Nejjari, Boutrous Khoury, Vicenç Puig, Joseba Quevedo, Josep Pascual, Sergi de Campos

**Affiliations:** 1 Advanced Control Systems Group, Automatic Control Department, Edifici Gaia Campus de Terrassa, Universitat Politècnica de Catalunya, Rambla Sant Nebridi, 22, 08222 Terrassa, Spain; 2 Institut de Robòtica i Informàtica Industrial (CSIC-UPC), Llorens i Artigas, 4-6, 08028 Barcelona, Spain; 3 ADASA Sistemas, S.A.U. C/Ignasi Iglesias, 217, 08820 El Prat de Llobregat, Spain

**Keywords:** economic model predictive control, linear parameter varying modelling, wastewater treatment process

## Abstract

This work proposes an economic model predictive control (EMPC) strategy in the linear parameter varying (LPV) framework for the control of dissolved oxygen concentrations in the aerated reactors of a wastewater treatment plant (WWTP). A reduced model of the complex nonlinear plant is represented in a quasi-linear parameter varying (qLPV) form to reduce computational burden, enabling the real-time operation. To facilitate the formulation of the time-varying parameters which are functions of system states, as well as for feedback control purposes, a moving horizon estimator (MHE) that uses the qLPV WWTP model is proposed. The control strategy is investigated and evaluated based on the ASM1 simulation benchmark for performance assessment. The obtained results applying the EMPC strategy for the control of the aeration system in the WWTP of Girona (Spain) show its effectiveness.

## 1. Introduction

Biological wastewater treatment plants (WWTPs) are complex nonlinear systems with large variations in their flow rates and feed concentrations. These plants have to be operated continuously taking care of strict environmental regulations. Thus, the use of advanced control strategies becomes necessary to make them more efficient.

The most widely used biological wastewater treatment is the activated sludge process (ASP). In the ASP, microorganisms are mixed with wastewater. The pollutants of the wastewater constitute the nutrient of the microorganisms. As the organisms feed on the organic pollutants in the wastewater, the pollutants are converted to more organisms, biomass, and some by-products. Following an adequate amount of treatment time, the mixture of microorganisms and wastewater, the mixed liquor flows from the aeration tank to a clarifier or settler where the sludge is separated from the treated water. Some of the settled sludge is continuously recirculated from the clarifier to the aeration tank to ensure the maintenance of adequate amounts of microorganisms in this tank. The microorganisms are again mixed with incoming wastewater where they are reactivated to consume organic nutrients. There are five major groups of microorganisms generally found in the aeration basin of the activated sludge process: (i) aerobic bacteria responsible for removing the organic nutrients, (ii) protozoa to remove and digest dispersed bacteria and suspended particles, (iii) metazoa to dominate longer age systems and clarify effluent, (iv) filamentous bacteria or bulking sludge, which are present when operating conditions change, (v) algae and fungi, which are photosynthetic organisms that are present with pH changes and older sludge.

The majority of the culture is mixed and reused with inlet wastewater to keep high reaction rates and sludge age characteristics. In particular, nitrogen is eliminated as follows: First, ammonium is oxidized producing nitrate under nitrification in the aerobic step. The nitrate that is produced is then converted into nitrogen gas by means of denitrification in the anoxic step. Thus, the control of aeration is very important because a low amount of dissolved oxygen can cause the biomass death. On the other hand, an excess of dissolved oxygen could cause the sludge to settle insufficiently. Moreover, because 60% to 80% of the global energy consumption is due to aeration and the operating costs of a WWTP [1], an excessive aeration is not desirable regarding economic efficiency.

The models that are usually considered for characterizing the WWTP processes are the ones developed by the International Association on Water Quality (IAWQ) known as Activated Sludge Models (ASMs) [2].

In this paper, optimal economic operation of the aeration system is considered to improve the efficiency and reliability of an ASP with intermittent aeration, which is used for the removal of nitrogen from domestic wastewater. The objective of the control is to design an aeration strategy (air-on and air-off periods) which minimizes the energy dissipated by the aeration system, with adherence to the limits of the effluent requirements and the operating constraints. The implementation of optimal operation strategies is therefore interesting because WWTPs face the challenge of treating water properly albeit ensuring the minimization of operational costs. This has been the driving force for the active research in the development of advanced control techniques and hierarchical control schemes to improve the operation of the WWTPs, see for example [3,4].

Model predictive control (MPC) has been the most successful advanced control approach applied to control WWTPs. This is due to the fact that MPC controllers allow in a straightforward manner the different operational requirements, the multivariate nature of the control problem (that could even include delay) and directly handling constraints on the control inputs, system outputs and/or internal states [5]. It can also include disturbance prediction, allowing to anticipate the appropriate control actions (feedforward) to achieve optimal performance according to defined criteria in the cost function, which can include different quality criteria and operational costs. Adjusting the MPC control strategy is carried out by suitable manipulating prioritization of different objectives of the performance index that could also include the use of soft constraints. In this way, MPC has become an attractive control strategy for a considerable number of WWTP applications in the last few years. Some examples of MPC control of WWTP can be found in [6,7]. In [6], a benchmarking of different hierarchical control structures for WWTPs that combines static and dynamic real-time optimization (RTO) and nonlinear model predictive control (NMPC) is presented. In [8], a procedure to find the best controlled variables in an economic sense for the activated sludge process in a wastewater treatment plant, despite the large load disturbances, is introduced.

Classical MPC formulation considers pre-established set points, and the objective functions related with error and energy effort have quadratic forms [5]. However, the determination of optimal and reachable reference set points in real time is not an easy task because of the existence of disturbances, set-point changes, time-varying parameters and model uncertainties, among others. This constitutes one of the main limitations of classical MPC. To remedy this issue, real-time optimizers (RTO) or steady-state target optimizers (SSTO) are used to pre-compute the reference set- points at a supervisory layer in the control hierarchy. Then, these pre-computed set points are sent to the lower layer, where a classical MPC behaves as a regulatory controller, forcing the process to follow the desired set points. However, in spite of the use of an RTO, not reachable trajectories might be generated because of the appearance of unexpected disturbances or set points variations, among others. Moreover, there is a delay between the different layers, because the lower layer receives the reference set points determined from the upper layer before its execution. These problems can be avoided by using economic MPC (EMPC) that optimizes process performance directly (e.g., by means of economic objective functions), eliminating the need of generating reachable reference set points [9]. The first results of the application of EMPC to DO concentration control in WWTPs have been presented in a previous work from the authors [10] considering the nonlinear model of the plant. However, this leads to a nonlinear optimization problem.

Alternatively, this paper proposes an EMPC strategy using the linear parameter-varying (LPV) framework to optimize the effluent quality and minimize the operational cost of a WWTP under operating and physical constraints. The objective is to minimize the energy used by the aeration system with the control of the dissolved oxygen (DO) concentrations in the aerated reactors and maintain the effluent concentration under the required limits. The proposed approach is based on real-time dynamic optimization methods. Optimization in MPC with nonlinear models presents a non-convex problem which is computationally demanding, especially when dealing with large-scale plants with complex dynamics such as the WWTP. Thus, the LPV framework allows the embedding of these nonlinearities in scheduling variables, which are functions of system states (i.e., qLPV). This allows obtaining a pseudo-linear model which is linear in state space but nonlinear in the parameter space and deriving a less demanding convex MPC optimization problem, since convex quadratic optimization tools can be applied. The stability and recursive feasibility of MPC with LPV models has been studied (see [11] for a review of the recent results). The application of dynamic optimization methods requires a sufficiently accurate mathematical model describing the wastewater treatment process. The present work uses the Activated Sludge Model No. 2 (ASM2) [12]. To illustrate the proposed approach a WWTP located in Girona (Spain) is considered as a case study.

In Section 2, the WWTP is described and modeled using a reduced ASM2 model, which is then represented in a qLPV form. The proposed EMPC strategy is introduced and described in Section 3, while the proposed MHE approach is presented in Section 4. The results are presented in Section 5, with simulation scenarios obtained from the application of the EMPC strategy on the Girona WWTP. Finally, some conclusions are given in Section 6.

## 2. WWTP Description and Modeling

### 2.1. WWTP Description

**Figure 1:**
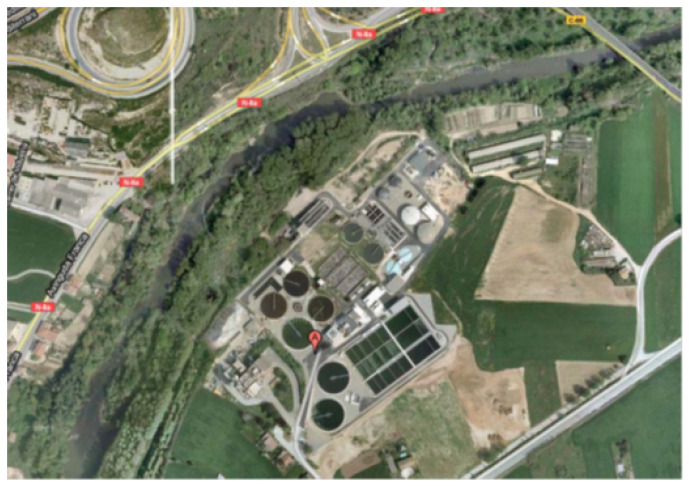
Girona Wastewater Treatment Plant.

The Girona WWTP is a biological treatment plant designed to treat the wastewater generated by 200,000 inhabitant equivalents with a medium daily inflow of 35,000 m^3^/d. The processes of the plant can be divided into two main treatment lines: water and sludge (see Figure 1). The water line is separated into three phases: pre-treatment, primary treatment and secondary treatment. The secondary treatment is designed to convert biodegradable, organic wastewater constituents and certain inorganic fractions into new cell mass and by-products. The plant uses an activated sludge system and has three lines composed of three main reactors that are divided into various compartments and three clarifiers. Each line is made of two anoxic reactors located at the beginning, three aerated tanks and an anoxic tank followed by an aerated one. With this configuration, the plant can nitrify and denitrify with great efficiency. The anoxic and aerobic tanks have volumes of 1335, 4554, 1929, and 1929 m${}^{3}$ for anoxic and 1929, 1276, and 1409 m${}^{3}$ for aerobic, respectively. Oxygen is supplied to aerated tanks by the aeration system, which delivers air to each of the aeration tanks. The wastewater and activated sludge are separated into three parallel secondary settlers. The volume of each secondary settler is approximately 5024 m${}^{3}$. The activated sludge is internally recirculated from the last aerobic zone to the anoxic tank (210% of influent waste). Additionally, the wastewater is recirculated from the secondary settlers to the anoxic tank (45 to 100% of influent waste).

**Figure 2:**
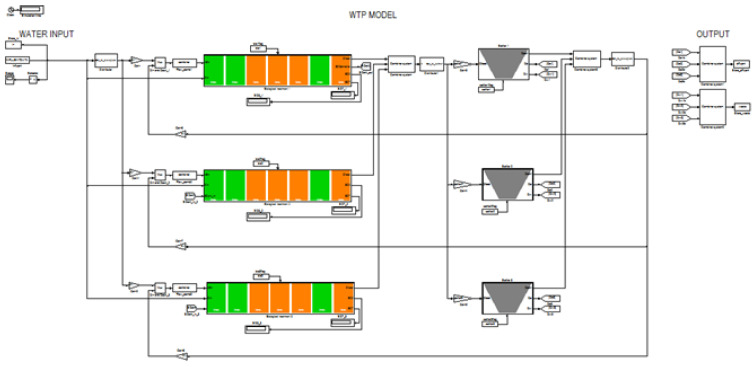
Layout of Girona WWTP.

Figure 2 shows a standard WWTP technological layout. The wastewater flow enters into the biological part after the mechanical treatment. The nutrient removal takes place in the activated sludge reactor through the biological treatment. The first zone in this treatment is anaerobic, where phosphorus is released. The mixed liquor internal recirculation originates from the anoxic zone. The denitrification occurs in the second zone. The activated sludge returned from the clarifiers bottom, and the internal recirculation from the aerobic zones end is directed toward the anoxic zone.

### 2.2. WWTP Modeling

The Benchmark Simulation Model (BSM1), developed within the framework of COST Actions 624 and 682 [2], has been adapted to represent the Girona WWTP (see Figure 1).

The Activated Sludge Model No. 1 (ASM1) describes the biological phenomena that takes place in the biological reactors, and it is supposed that no biological reactions take place in the settlers. Due to the complexity of the nonlinear model describing the different complex processes in the plant, various reduced models have been proposed in the literature [13,14,15] to aid in the online implementation of certain modern control schemes (e.g., MPC), which would have otherwise presented ill-conditioned or stiff numerical problems due to slow and fast dynamic interactions. In [15,16], one can see some successful implementations using reduced WWTP models in various areas of control applied to WWTPs. The reduced model as suggested in [14], which primarily involves certain simplification criteria for a reduced order of the rigorous high dimensional WWTP model, has been adapted to conditions representing the Girona WWTP. This basically involves the derivation of the reactor model based on mass balances of the wastewater species, which are generally expressed as follows:

Accumulation=Inflow−Outflow+Reaction

Validation of the reduced model considering data from the ASM1 and the reduced model has been undertaken in [14]. In simplifying the complex model, a systematic reduction process of the high-dimensional model considers some assumptions, with the principal conditions given as follows:

- The soluble ($S_{S}$) and particulate ($X_{S}$) organic compounds are aggregated as a single variable $X_{COD}$, the chemical oxigen demand (COD).
- Through reduction by time scale from the theory of singular pertubation, the slow dynamics of the variables $X_{I}$, $X_{BH}$ and $X_{BA}$ together with the soluble inert organic compounds **$\left(S_{I}\right)$** are excluded.
- Finally, simplification of complicated kinetic process, assumption of no alkalinity and separation of aerobic and anoxic conditions are considered.

Under these conditions, the resultant state variables of the reduced model are therefore the chemical oxygen demand ($X_{COD}$), the dissolved oxygen concentration, ($S_{O}$), heterotrophic biomass, $X_{BH}$, ammonia concentration ($S_{NH}$), nitrate concentration ($S_{NO}$) and autotrophic biomass ($X_{BA}$). The control of oxygen concentration ($S_{0}$) in the aerobic tanks is via the manipulation of the control input, the oxygen transfer coefficient $K_{La}\left(t\right)$.

The states and input vectors are thus given as:

$$x\left(t\right)={\left[X_{COD}\left(t\right),S_{O}\left(t\right),X_{BH}\left(t\right),S_{NH}\left(t\right),S_{NO}\left(t\right),X_{BA}\left(t\right)\right]}^{T}$$

$$u\left(t\right)=K_{La}\left(t\right)$$

The WWTP process is therefore described by the following dynamic equations of the reduced model:

(1) $$\begin{array}{ll} {\dot{X}}_{COD}\left(t\right) & =\frac{1}{Y_{h}}\left[\theta_{1}\left(t\right)+\theta_{2}\left(t\right)\right]+\left(1-f_{p}\right)\left(\theta_{4}\left(t\right)+\theta_{5}\left(t\right)\right)+\vartheta_{1}\left(t\right), \end{array}$$

(2) $$\begin{array}{cc} {\dot{S}}_{O}\left(t\right) & =\frac{Y_{h}-1}{Y_{h}}\theta_{1}\left(t\right)+\frac{Y_{a}-4.57}{Y_{a}}\theta_{3}\left(t\right)+\vartheta_{2}\left(t\right), \end{array}$$

(3) $$\begin{array}{cc} {\dot{S}}_{NH}\left(t\right) & =-i_{xb}\left[\theta_{1}\left(t\right)+\theta_{2}\left(t\right)\right]-\left[i_{xb}+\frac{1}{Y_{a}}\right]\theta_{3}\left(t\right)+\left(i_{xb}-f_{p}i_{xp}\right)\left[\theta_{4}\left(t\right)+\theta_{5}\left(t\right)\right]+\vartheta_{3}\left(t\right), \end{array}$$

(4) $$\begin{array}{cc} {\dot{S}}_{NO}\left(t\right) & =\frac{Y_{h}-1}{2.86Y_{h}}\theta_{2}\left(t\right)+\frac{1}{Y_{a}}\theta_{3}\left(t\right)+\vartheta_{4}\left(t\right), \end{array}$$

(5) $$\begin{array}{cc} {\dot{X}}_{BH}\left(t\right) & =\theta_{1}\left(t\right)+\theta_{2}\left(t\right)-\theta_{4}\left(t\right)+\vartheta_{5}\left(t\right), \end{array}$$

(6) $$\begin{array}{cc} {\dot{X}}_{BA}\left(t\right) & =\theta_{3}\left(t\right)-\theta_{5}\left(t\right)+\vartheta_{6}\left(t\right). \end{array}$$

where

$$\begin{array}{cc} \theta_{1}\left(t\right) & =\mu_{h}\frac{X_{COD}\left(t\right)}{K_{COD}+X_{COD}\left(t\right)}\frac{S_{O}\left(t\right)}{K_{OH}+S_{O}\left(t\right)}X_{BH}\left(t\right)\\ \theta_{2}\left(t\right) & =\mu_{h}\eta_{NOg}\frac{X_{COD}\left(t\right)}{K_{COD}+X_{COD}\left(t\right)}\frac{S_{NO}\left(t\right)}{K_{NO}+S_{NO}\left(t\right)}\frac{K_{OH}}{K_{OH}+S_{O}\left(t\right)}X_{BH}\left(t\right)\\ \theta_{3}\left(t\right) & =\mu_{a}\frac{S_{NH}\left(t\right)}{K_{NH,A}+S_{NH}\left(t\right)}\frac{S_{O}\left(t\right)}{K_{O,A}+S_{O}\left(t\right)}X_{BA}\left(t\right)\\ \theta_{4}\left(t\right) & =b_{H}X_{BH}\left(t\right)\\ \theta_{5}\left(t\right) & =b_{A}X_{BA}\left(t\right) \end{array}$$

With the flow rate given as $Q_{in}\left(t\right)$, $V_{o}$ as the volume of the aerobic tank and considering that $S_{0_{in}}\left(t\right),S_{NO_{in}}\left(t\right),X_{BA_{in}}\left(t\right)$ are equal to zero. $\vartheta_{1}\left(t\right),\vartheta_{2}\left(t\right),\cdots ,\vartheta_{6}\left(t\right)$ are given as follows:

$$\begin{array}{cc} \vartheta_{1}\left(t\right) & =\frac{Q_{in}\left(t\right)}{V_{o}}\left[X_{COD_{in}}\left(t\right)-X_{COD}\left(t\right)\right]\\ \vartheta_{2}\left(t\right) & =\frac{Q_{in}\left(t\right)}{V_{o}}\left[-S_{O}\left(t\right)\right]+K_{La}\left(t\right)\left[S_{O_{sat}}-S_{O}\left(t\right)\right]\\ \vartheta_{3}\left(t\right) & =\frac{Q_{in}\left(t\right)}{V_{o}}\left[S_{NH_{in}}\left(t\right)-S_{NH}\left(t\right)\right]\\ \vartheta_{4}\left(t\right) & =\frac{Q_{in}\left(t\right)}{V_{o}}\left[-S_{NO}\left(t\right)\right]\\ \vartheta_{5}\left(t\right) & =\frac{Q_{in}\left(t\right)}{V_{o}}\left[X_{BH_{in}}\left(t\right)-\frac{f_{w}\left(1+f_{r}\right)}{f_{r}+f_{w}}X_{BH}\left(t\right)\right]\\ \vartheta_{6}\left(t\right) & =\frac{Q_{in}\left(t\right)}{V_{o}}\left[X_{BA}\left(t\right)-\frac{f_{w}\left(1+f_{r}\right)}{f_{r}+f_{w}}X_{BA}\left(t\right)\right] \end{array}$$

where $Y_{H}$, $Y_{A}$, $f_{r}$, $f_{w}$, $b_{h}$, $b_{A}$, $i_{xb}$, and $f_{p}$ are the stoichiometric parameters and $\mu_{h}$, $K_{COD}$, $K_{OH}$, $\mu_{a}$, $K_{NH,A}$, and $K_{O,A}$ are the kinetic parameters.

### 2.3. LPV Representation of the WWTP

For ease of computational burden, the nonlinear reduced model is represented in a LPV form which involves the embedding of nonlinearities in varying parameters, resulting in a linear representation in state space. This procedure offers benefits when applied to MPC over its nonlinear MPC [15] and linear MPC [17] counterparts as applied on the WWTP by providing a faster run time and the avoidance of numerical problems with respect to the former and the ability to operate in a wide range of operating points with regard to the latter. The nonlinear model in this case is defined by linear systems at each time instance based on some time-varying parameters $\sigma \left(t\right)\in R^{n_{\sigma }}$, with an assumption that the parameters σ(t) are not known a priori but can be measured or estimated online [18]. The dynamic behavior of the LPV model is therefore described as:

(7) $$\dot{x}\left(t\right)=A\left(\sigma \left(t\right)\right)x\left(t\right)+B\left(\sigma \left(t\right)\right)u\left(t\right)$$

(8) y(t)=C(σ(t))x(t)+D(σ(t))u(t)

where $x\left(t\right)\in R^{n_{x}}$ and $u\left(t\right)\in R^{n_{u}}$ are the states and inputs, respectively, with $y\left(t\right)\in R^{n_{y}}$ as the measured signals. A(σ(t)), B(σ(t)), C(σ(t)) and D(σ(t)) are time-varying matrices of appropriate dimensions that are affine in $\sigma \left(t\right)\in R^{n_{\sigma }}$. In the quasi LPV case, the scheduling parameters are dependent on measured signals, $y_{s}\left(t\right)\in R^{k}\subset y\left(t\right)\in R^{n_{y}}$, such that

$$\sigma \left(t\right)=f(y_{s}\left(t\right))$$

where $f:R^{k}\mapsto R^{n_{\sigma }}$ is a continuous mapping [19]. With observed states and exogenous inputs (w(t)), nonlinearities involving the system states can be “hidden” in the varying parameters, $\sigma (t,y_{s}\left(t\right),w\left(t\right))$.

Therefore, from the generic nonlinear form

(9) $$\begin{array}{ccc} \; & \dot{x}\left(t\right)=f\left(x\left(t\right),u\left(t\right),w\left(t\right)\right)\\ \; & y(t)=g(x(t),u(t)) & \; \end{array}$$

a linear quadruple $(A\left(\sigma \left(t,y_{s}\left(t\right),w\left(t\right)\right)\right)$, $B(\sigma \left(t,y_{s}\left(t\right),w\left(t\right)\right))$, $C(\sigma \left(t,y_{s}\left(t\right),w\left(t\right)\right)),and$ $D(\sigma \left(t,y_{s}\left(t\right),w\left(t\right)\right))$ estimate is formulated and incorporated into the EMPC for a convex optimization problem. In the following, the function $\sigma (t,y_{s}\left(t\right),w\left(t\right)))$ will simply be represented as σ(t). The choice of scheduling parameters considering the origin of nonlinearities in the reduced model (1) are

$$\begin{array}{cc} \; & \sigma_{1}\left(t\right)=Q_{in}\left(t\right),\;\;\sigma_{2}\left(t\right)=\frac{X_{COD}\left(t\right)}{K_{COD}+X_{COD}\left(t\right)}\frac{X_{BH}\left(t\right)}{K_{OH}+S_{O}\left(t\right)},\\ \; & \sigma_{3}\left(t\right)=\frac{X_{COD}\left(t\right)}{K_{COD}+X_{COD}\left(t\right)}\frac{S_{NO}\left(t\right)}{K_{NO}+S_{NO}\left(t\right)}\frac{K_{OH}}{K_{OH}+S_{O}\left(t\right)},\\ \; & \sigma_{4}\left(t\right)=\frac{1}{K_{OA}+S_{O}\left(t\right)}\frac{S_{NH}\left(t\right)}{K_{NH}+S_{NH}\left(t\right)}X_{BH}\left(t\right),\;\;\sigma_{5}\left(t\right)=S_{O}\left(t\right). \end{array}$$

The dynamic LPV model is thus given as:

(10) $$\dot{x}=A\left(\sigma \left(t\right)\right)x\left(t\right)+B\left(\sigma \left(t\right)\right)u\left(t\right)+Ew\left(t\right).$$

with the time-varying matrices, A(σ(t)), B(σ(t)), and time-invariant disturbance matrix *E* as:

$$A\left(\sigma \left(t\right)\right)=\left[\begin{array}{cccccc} a_{11}\left(t\right) & 0 & 0 & 0 & a_{15}\left(t\right) & a_{16}\\ 0 & a_{22}\left(t\right) & 0 & 0 & a_{25}\left(t\right) & 0\\ 0 & a_{32}\left(t\right) & 0 & 0 & a_{35}\left(t\right) & a_{36}\\ 0 & a_{42}\left(t\right) & 0 & 0 & a_{45}\left(t\right) & 0\\ 0 & 0 & 0 & 0 & a_{55}\left(t\right) & 0\\ 0 & a_{62}\left(t\right) & 0 & 0 & 0 & a_{66}\left(t\right) \end{array}\right],$$

$$B\left(\sigma \left(t\right)\right)=\left[\begin{array}{c} 0\\ b_{12}\left(t\right)\\ 0\\ 0\\ 0\\ 0 \end{array}\right]\;\text{and}\;E=\frac{1}{V_{O}}\left[\begin{array}{ccc} 1 & 0 & 0\\ 0 & 0 & 0\\ 0 & 1 & 0\\ 0 & 0 & 0\\ 0 & 0 & 1\\ 0 & 0 & 0 \end{array}\right].$$

where

$\begin{array}{cc} \; & a_{11}\left(t\right)=\frac{\sigma_{1}\left(t\right)}{V_{o}},\;\;a_{15}\left(t\right)=-\frac{\mu_{h}}{Y_{h}}\sigma_{2}\left(t\right)+\left(1-f_{p}\right)b_{h}-\frac{\mu_{h}\eta_{NOg}}{Y_{h}}\sigma_{3}\left(t\right),\;\;a_{16}=\left(1-f_{p}\right)b_{a},\\ \; & a_{22}\left(t\right)=-\frac{\sigma_{1}\left(t\right)}{V_{O}}-\frac{4.57-Y_{a}}{Y_{a}}\mu_{a}\sigma_{4}\left(t\right),\;\;a_{25}\left(t\right)=\frac{Y_{h}-1}{Y_{h}}\mu_{h}\sigma_{2}\left(t\right)\\ & a_{32}\left(t\right)=-\left(i_{xb}+\frac{1}{Y_{a}}\right)\mu_{a}\sigma_{4}\left(t\right),\;\;a_{35}\left(t\right)=\left(i_{xb}-f_{p}i_{xp}\right)b_{h}-i_{xb}\mu_{h}\sigma_{2}\left(t\right)-\left(i_{xb}\mu_{h}\sigma_{3}\left(t\right)\right),\\ \; & a_{36}=\left(i_{xb}-f_{p}i_{xb}\right)b_{a},\;\;a_{42}\left(t\right)=\frac{1}{Y_{a}}\mu_{a}\sigma_{4}\left(t\right),\;\;a_{45}\left(t\right)=\frac{Y_{h}-1}{2.86Y_{h}}\mu_{h}\eta_{NOg}\sigma_{3}\left(t\right),\\ \; & a_{55}\left(t\right)=\mu_{h}\sigma_{2}\left(t\right)-b_{h}-\left(\frac{\sigma_{1}\left(t\right)}{V_{O}}-\frac{f_{w}\left(1+f_{r}\right)}{f_{r}+f_{w}}\right)-b_{a},\;\;a_{62}\left(t\right)=\mu_{a}\sigma_{4}\left(t\right),\\ \; & a_{66}\left(t\right)=\frac{\sigma_{1}\left(t\right)}{V_{O}}\left(-\frac{f_{w}\left(1+f_{r}\right)}{f_{r}+f_{w}}-1-b_{a}\right),\;\;b_{12}\left(t\right)=S_{sat}-\sigma_{5}\left(t\right). \end{array}$

The input concentrations are

$$w\left(t\right)={\left[\begin{array}{ccc} Q_{in}\left(t\right)X_{{COD}_{in}}\left(t\right) & Q_{in}\left(t\right)S_{{NH}_{in}}\left(t\right) & Q_{in}\left(t\right)X_{{BH}_{in}}\left(t\right) \end{array}\right]}^{T}.$$

**Remark** **1.** *In this work, it is assumed that all the concentrations are measured online, but it must be noted that in practice, not all the concentrations, such as, e.g., $X_{{COD}_{in}}$ can be measured.*

## 3. EMPC of a WWTP

### 3.1. Operational Goals

The immediate control goal of a WWTP is to meet water quality levels established by regulators while operating efficiently by reducing operational cost. As discussed in the introduction, predictive control techniques may be used to compute strategies which achieve this goal while at the same time optimizing the system performance in terms of different operational indices. To achieve this objective, the control of dissolved oxygen concentration as well as nitrates within certain limits is necessary. The MPC presents the advantage of being a non-conservative control strategy, such that in periods of low influents, with a minimal level of pollutants, the effluent quality can be achieved by regulating the levels of $S_{0}$ and $S_{NO}$ below the stipulated reference point to avoid waste of energy. Subsequently, during periods of high influents levels, it is then important to meet the predefined set points to reduce pollutants, avoiding the violation of the standard effluent quality set by authorities [15]. In this work, a PI-EMPC control strategy is employed: PI designed by authors of the BSM1 for the regulation of $S_{NO}$ and a designed EMPC for the control of $S_{O}$ in the aeration tank. In the proposed LPV EMPC, the following objectives are then considered:

- **Economic costs.** The main economic costs associated with WWTP are primarily due to treatment and electricity costs. Water through the WWTP involves important electricity costs in pumping stations in charge of internal and external water recirculations as well as aeration in the aerobic tanks. In our case, only the aeration energy is considered with an objective of minimizing the cost associated with supply of oxygen for controlled culture growth. The performance index is described as follows
  (11) $$J_{eco}\left(k\right)=\frac{S_{o_{sat}}}{1800}V_{o}K_{La}\left(k\right)\left[\frac{\mathrm{kwh}}{\mathrm{day}}\right].$$
- **DO concentration control.** In order to control the $S_{o}$ within some bounds in the EMPC during the aeration process, slack variables are introduced in the optimization problem, which seek to penalize the dissolved oxygen states, such that they are maintained in a range to maintain effluent quality. Selecting slack variables, $(\lambda^{+}>0$ and $\lambda^{-}>0)$, additional terms of soft constraints (see (16c) and (16d)) and a quadratic objective index are introduced with $x_{sp}$ as the selected DO concentration value. The introduction of the slack variables ensures that the DO concentration varies within a boundary around $x_{sp}$ aided by the appropriate selection of weights in the objective function. The performance index is thus given as
  (12) $$J_{\lambda }\left(k\right)={∥\lambda \left(k\right)∥}_{2}^{2},$$
  where $\lambda \left(k\right)={\left[\lambda^{-}\left(k\right),\lambda^{+}\left(k\right))\right]}^{T}$.
- **Smooth set points for equipment conservation.** The operation of WWTP and main valves and pumps usually requires smooth flow set-point variations. To obtain such a smoothing effect, the proposed MPC controller includes a third term in the objective function to penalize the control signal variation between consecutive time intervals. This term is expressed as
  (13) $$J_{smo}\left(k\right)=\Delta u{\left(k\right)}^{T}W_{u}\Delta u\left(k\right).$$
  Therefore, the performance function *J* considering the aforementioned control objectives has the form
  (14) $$J=w_{1}\sum_{k=0}^{H_{p}-1}J_{eco}\left(k\right)+w_{2}\sum_{k=0}^{H_{p}-1}J_{smo}\left(k\right)+w_{3}\sum_{k=1}^{H_{p}}J_{\lambda }\left(k\right).$$

### 3.2. Control Strategy Computation

The control strategy is determined by the computation of an optimal sequence of control actions for a prediction horizon, $H_{p}$.

(15) $${\tilde{u}}_{k}={\left(u(\left.k\right|j)\right)}_{j=0}^{H_{p-1}}=\left(u\left(\left.k\right|0\right),u\left(\left.k\right|1\right),\cdots ,u\left(\left.k\right|H_{p-1}\right)\right).$$

We solve at each time instance *k*, the following optimal control problem with initial state obtained from measurements (or state estimation) of the dynamics WWTP model and prediction in the MPC loop with the qLPV plant model (10),

(16a) $$\begin{array}{c} \min_{{\tilde{u}}_{k}}J\left({\tilde{u}}_{k},k\right) \end{array}$$

subject to

(16b) $$\begin{array}{c} x\left(i+1|k\right)=A\left(\sigma \left(k\right)\right)x\left(i|k\right)+B\left(\sigma \left(k\right)\right)u\left(i|k\right)+Ew\left(i|k\right)\;i=0,\cdots ,H_{p}-1, \end{array}$$

(16c) $$\begin{array}{c} x_{s_{o}}\left(i|k\right)>=x_{sp}+\lambda^{+}\left(i|k\right),\;\;\;i=1,\cdots ,H_{p}, \end{array}$$

(16d) $$\begin{array}{c} x_{s_{o}}\left(i|k\right)<=x_{sp}-\lambda^{-}\left(i|k\right),\;\;\;i=1,\cdots ,H_{p}, \end{array}$$

(16e) $$\begin{array}{cccc} & \;u\left(i|k\right)\in U\;i=0,\cdots ,H_{p}-1, & & \end{array}$$

(16f) $$\begin{array}{cccc} & x\left(i|k\right)\in X\;\;\;i=1,\cdots ,H_{p}, & & \end{array}$$

(16g) $$\begin{array}{cccc} & y\left(i|k\right)\in Y\;\;\;j=0,\cdots ,H_{p}, & & \end{array}$$

(16h) $$\begin{array}{cc} & \lambda^{+}\left(i|k\right),\lambda^{-}\left(i|k\right)>=0 \end{array}$$

where $x_{s_{o}}$ is the dynamic state representing the soluble oxygen. (16c–f) are described by the box constraints:

(17) $$\begin{array}{c} U=\left\{\left.u\in R^{n_{u}}\right|u^{\min}\leq u\leq u^{\max}\right\},\\ X=\left\{\left.x\in R^{n_{x}}\right|x^{\min}\leq x\leq x^{\max}\right\},\\ Y=\left\{\left.y\in R^{n_{y}}\right|y^{\min}\leq y\leq y^{\max}\right\}. \end{array}$$

which are determined from the maximum residual concentrations imposed in order to cope with the European Union effluent standards on chemical oxygen demand COD, suspended solids $S_{S}$ and total nitrogen $T_{N}$:

$$COD⩽COD_{max}=125\;gm^{-3},$$

$$S_{S}⩽S_{Smax}=35\;gm^{-3},$$

$$T_{N}⩽T_{Nmax}=10\;gm^{-3}.$$

The first control action of the sequence $u(\left.k\right|0)$ is applied to the WWTP plant to obtain the system measurements and/or MHE estimated states, which are then used in the succeeding optimization problem, resulting in a recursive procedure. Not all the state variables are measured as stated earlier; the moving horizon estimator (MHE), which is the dual of the MPC controller, estimates the unmeasurable states.

## 4. Moving Horizon Estimation

**Figure 3:**
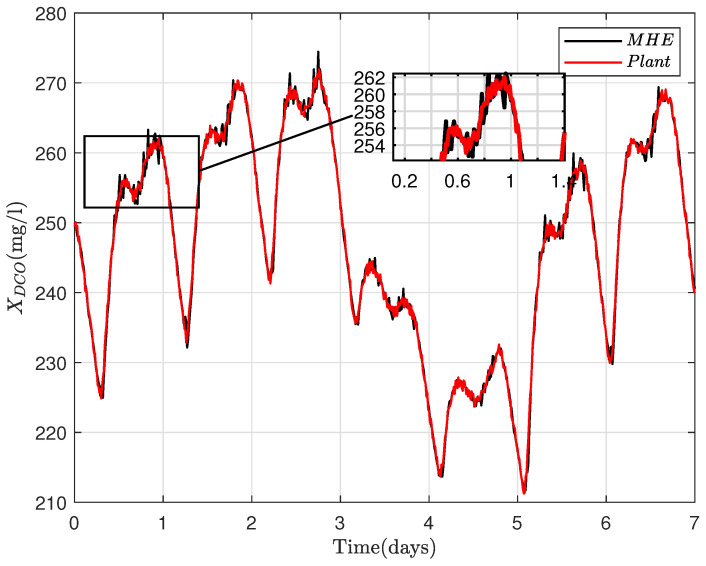
MHE estimate of oxygen demand concentration ($X_{COD}$) for 7 days.

**Figure 4:**
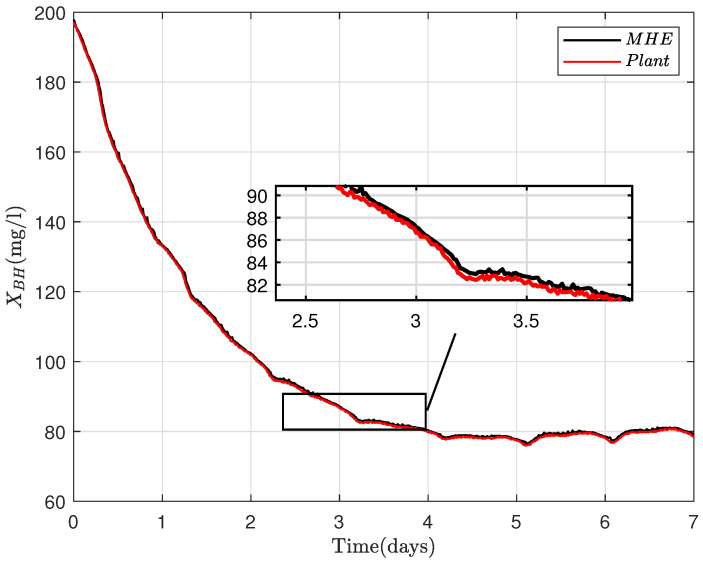
MHE estimate of heterotrophic biomass ($X_{BH}$) for 7 days.

**Figure 5:**
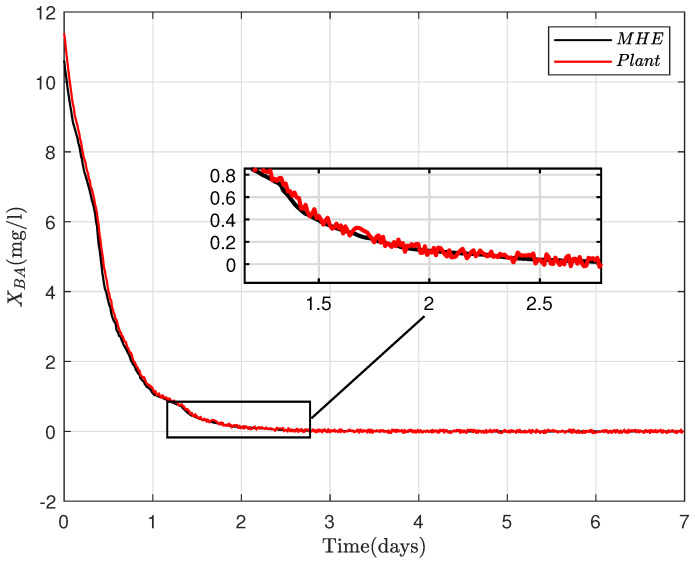
MHE estimate of autotrophic biomass ($X_{BA}$) for 7 days.

Since some states cannot be measured online in the operation of the WWTP, a design of an estimator, in our case the MHE, is necessary for the prediction of system outputs, bearing in mind that apart from purposes of feedback control, the quasi-LPV formulation relies on information of the system states for the model construction. By solving a constrained optimization problem, the MHE utilizes a limited *N*-prediction horizon of past measurements through an error minimization scheme aided by information of the system model in a prediction window to estimate the system states. The optimization problem is therefore set up with the discretized plant model as:

(18) $$\begin{array}{cccc} \; & \min_{{\left\{\hat{x}\left(i|k\right)\right\}}_{i=-N}^{0}}\; & \; & {\left(\hat{x}\left(-N|k\right)-x_{o}\right)}^{T}P_{o}\left(\hat{x}\left(-N|k\right)-x_{o}\right)+\sum_{i=-N}^{k}\left(\epsilon {\left(i|\right)}^{T}Q\epsilon \left(i|k\right)+s{\left(i|k\right)}^{T}Rs\left(i|k\right)\right)\\ \; & s.t.\; & \; & \hat{x}\left(i+1|k\right)=A\left(\sigma \left(i|k\right)\right)\hat{x}\left(i|k\right)+B\left(\sigma \left(i|k\right)\right)u\left(i|k\right)+Ew\left(i|k\right)+\epsilon \left(i|k\right)\;i=-N,\cdots ,-1,\\ \; & \; & & y(i|k)=Cx(i|k)+s(i|k),\\ \; & \; & & {\hat{x}}_{k}\in X. \end{array}$$

where $R=R^{T}\in R^{n_{y}\times n_{y}}>0$, $Q=Q^{T}\in R^{n_{x}\times n_{x}}\geq 0$ and $P_{o}=P_{o}^{T}\in R^{n_{x}\times n_{x}}\geq 0$ are the weighting matrices that are defined according to uncertainty levels induced respectively by the noise, disturbance and unknown initial conditions ($x_{o}$). X bounds the estimated states. At every iteration, *N* sets of control inputs, ${\left\{u\left(i|k\right)\right\}}_{i=-N}^{-1}\in R^{n_{u}\times N}$, measurements ${\left\{y\left(i|k\right)\right\}}_{i=-N}^{-1}\in R^{n_{y}\times N}$ and *N* sets of LPV matrices ${\left\{A_{i}\right\}}_{i=-N}^{-1}\in R^{(n_{x}\times n_{x})N}$, ${\left\{B_{i}\right\}}_{i=-N}^{-1}\in R^{(n_{x}\times n_{u})N}$ are taken as inputs into the optimization problem to predict the state sequence ${\left\{\hat{x}\left(i|k\right)\right\}}_{i=-N}^{0}\in R^{n_{x}\times \left(N+1\right)}$ by solving the dynamical optimization problem (18). The last element of the sequence ${\left\{\hat{x}\left(i|k\right)\right\}}_{i=-N}^{0}$ is subsequently chosen as the estimated states, the measurements and inputs are then discarded, and the procedure is repeated. The ammonia concentration $\left(S_{NH}\right)$, nitrate concentration $\left(S_{NO}\right)$ and the soluble oxygen $\left(S_{o}\right)$ are supposedly measurable; therefore, the MHE is designed for the estimation of ${\left[X_{COD},X_{BH},X_{BA}\right]}^{T}$ as shown in Figure 3, Figure 4 and Figure 5 .

## 5. Simulation Results

### 5.1. LPV EMPC Implementation Details

To illustrate LPV EMPC approach presented in this paper, the Girona WWTP case study presented in Section 2 is used. The constituents of the influent wastewater of Girona WWTP varies during the day between the following bounds:

- Qin (between 10,000–35,000 m${}^{3}$/d);
- COD (between 400–650 mg/L);
- DBO (175–225 mg/L); and
- Nitrogen (between 40–65 mg/L).

**Figure 6:**
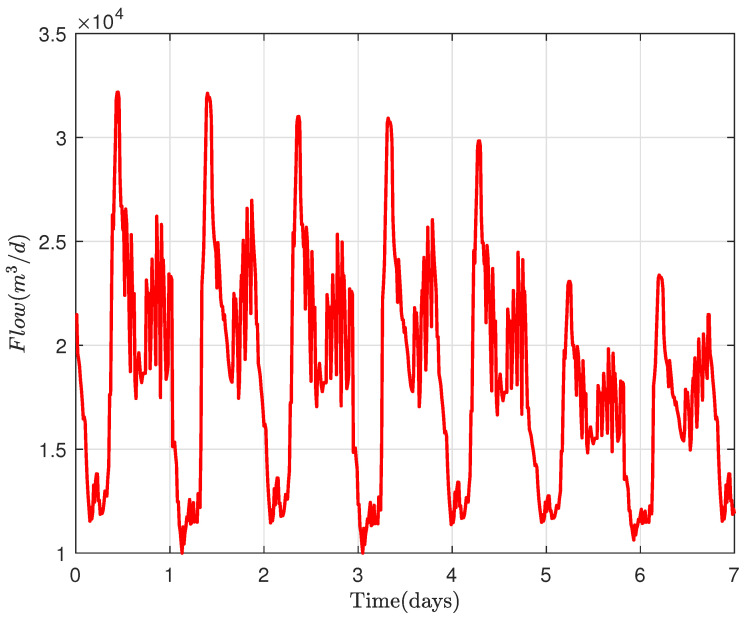
WWTP inflow.

The inflow of Girona WWTP is shown in Figure 6.

With a quasi-linear approximation of the nonlinear WWTP via the LPV representation, the constrained optimization problem (16) is solved using quadratic programming formulation using the CPLEX^®^ solver in MATLAB^®^ on an Intel Core i7, 8 GB of RAM PC. A sampling time of 15 min and a prediction horizon of 6 h is chosen for simulation. The process is simulated for 7 days in a Simulink environment representing the dynamics of the Girona WWTP, as shown in Figure 1.

Using the weights $w_{i}$ associated with the multiobjective EMPC cost function, (14) is tuned using the procedure as performed in [20,21] with the aim of maintaining the quality of the exit water at some levels within the current regulations regardless of the entry at a minimum cost.

Some control scenarios are selected to show different behaviors of the proposed scheme by altering $X_{sp}$ and manipulating weights $w_{i}$, ideally to illustrate the different actions of aeration corresponding to different dissolved oxygen requirements for a quality effluent.

### 5.2. First Scenario

**Figure 7:**
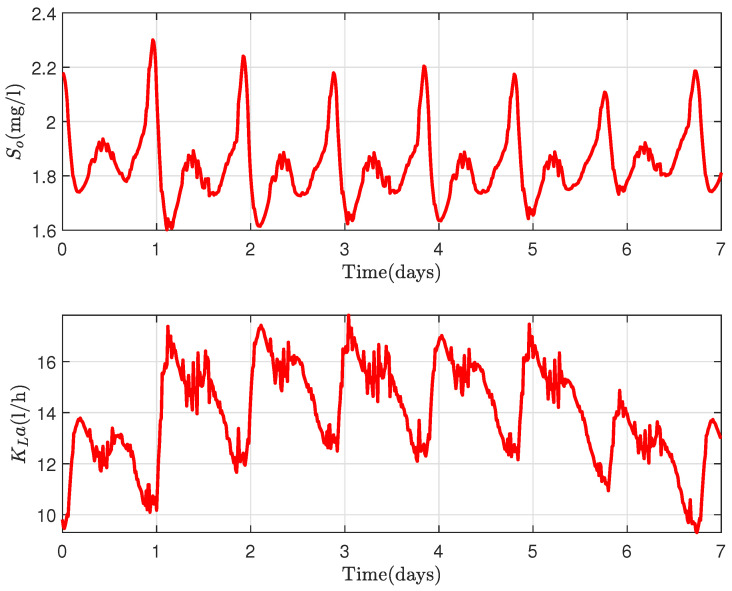
(**Above**): DO concentraton variation. (**Below**): Aeration flow for Scenario 1.

The first scenario consists of controlling the dissolved oxygen concentration in the exit of the biological treatment plant between the bounds (1.5,2.5). Figure 7 shows the dynamics of the DO concentration (above) and its corresponding aeration energy (below). The operation of the aeration, as stated in the preceding section, corresponds to the variation of the influents during the day; therefore, the DO concentration varies between the defined bounds in relation to the amount of pollutants at each time instance in the influents, which can be inferred from Figure 6.

### 5.3. Second Scenario

The second scenario also consists of controlling the DO concentration between the ranges of 0.5 to 1.2 mg/L with minimum aeration energy consumption.

**Figure 8:**
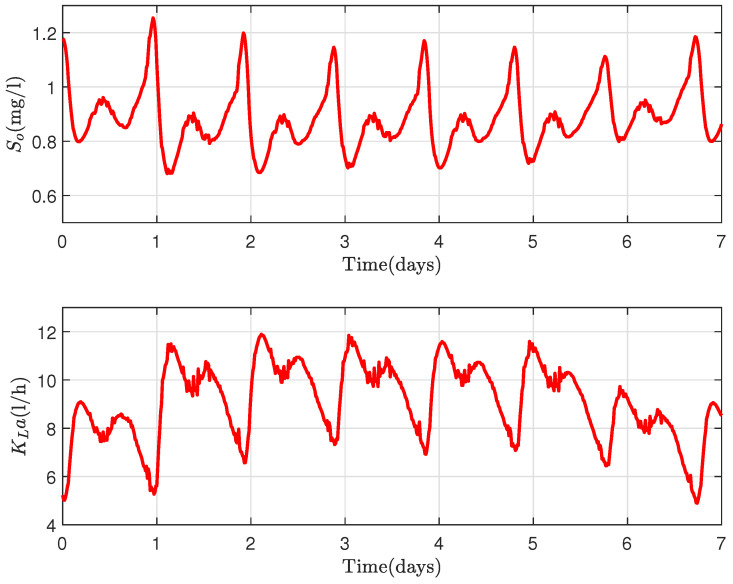
(**Above**): DO concentration variation and (**Below**): Aeration flow for Scenario 2.

**Figure 9:**
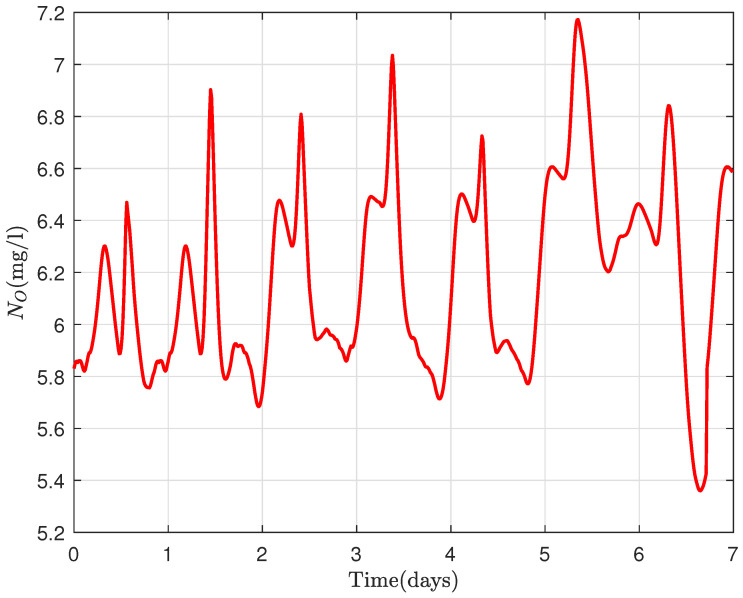
Nitrate concentration variation.

From Figure 8, a similar behavior of oxygen in the tanks as in the first scenario is realized with an expected less aeration energy, as less DO is required for treatment. The nitrates in the exit of the WWTP (Figure 9) range approximately between 5 and 7 mg/L.

## 6. Conclusions

In this paper, an LPV EMPC strategy for the control of dissolved oxygen concentration in the aerated reactors of a WWTP is proposed and applied to the Girona (Spain) case study. The proposed approach combines two improvements with respect to the existing approaches in the literature: First, differently from standard tracking MPC, the proposed EMPC strategy optimizes the economic performance of the plant instead of following some pre-established set points. Second, a reduced model of the WWTP is represented in a quasi-LPV form allowing the real-time implementation of the controller thanks to the use of quadratic programming optimization tools. If otherwise, the nonlinear model plant was used, nonlinear programming algorithms are required that usually prevent the real-time implementation because of the large computational time. Moreover, an LPV moving horizon state estimation scheme has also been proposed that allows the implementation of the LPV EMPC with the available sensors in the WWTP. The effectiveness of the proposed scheme has been illustrated in the considered case study with two scenarios aiming at keeping the DO within some bounds.

As future work, real testing in the WWTP plant will be conducted to further validate the performance of the proposed solution. Another issue to take into consideration is the application of the proposed methodology for aerobic conditions maintenance in sewer networks [22].

## Data Availability

All the required data are included in the paper.

## Abbreviations

The following abbreviations are used in this manuscript:

ASM	Active Sludge Model
ASP	Active Sludge Process
BSM	Benchmark Simulation Model
COD	Chemical Oxygen Demand
DO	Dissolved Oxygen
EMPC	Economic Model Predictive Control
qLPV	Quasi-Linear Parameter Varying
LPV	Linear Parameter Varying
MHE	Moving Horizon Estimator
MPC	Model Predictive Control
NEMPC	Nonlinear Economic Model Predictive Control
NMPC	Nonlinear Model Predictive Control
RTO	Real-Time Optimization
SSTO	Steady-State Target Optimizator
WWTP	Wastewater Treatment Plant
